# Flow-Through Electrochemical Biosensor for the Detection of *Listeria monocytogenes* Using Oligonucleotides

**DOI:** 10.3390/s21113754

**Published:** 2021-05-28

**Authors:** Cheryl M. Armstrong, Joe Lee, Andrew G. Gehring, Joseph A. Capobianco

**Affiliations:** United States Department of Agriculture, Agriculture Research Service, Eastern Regional Research Center, 600 East Mermaid Lane, Wyndmoor, PA 19038, USA; cheryl.armstrong@usda.gov (C.M.A.); joe.lee@usda.gov (J.L.); andrew.gehring@USDA.gov (A.G.G.)

**Keywords:** biosensor, rapid detection, foodborne pathogen, flow-through transducer, graphite felt, *Listeria monocytogenes*, *Listeria innocua*

## Abstract

Consumption of food contaminated by *Listeria monocytogenes* can result in Listeriosis, an illness with hospitalization rates of 94% and mortality rates up to 30%. As a result, U.S. regulatory agencies governing food safety retain zero-tolerance policies for *L. monocytogenes*. However, detection at such low concentrations often requires strategies such as increasing sample size or culture enrichment. A novel flow-through immunoelectrochemical biosensor has been developed for *Escherichia coli* O157:H7 detection in 1 L volumes without enrichment. The current work further augments this biosensor’s capabilities to (1) include detection of *L. monocytogenes* and (2) accommodate genetic detection to help overcome limitations based upon antibody availability and address specificity errors in phenotypic assays. Herein, the conjugation scheme for oligo attachment and the conditions necessary for genetic detection are laid forth while results of the present study demonstrate the sensor’s ability to distinguish *L. monocytogenes* DNA from *L. innocua* with a limit of detection of ~2 × 10^4^ cells/mL, which agrees with prior studies. Total time for this assay can be constrained to <2.5 h because a timely culture enrichment period is not necessary. Furthermore, the electrochemical detection assay can be performed with hand-held electronics, allowing this platform to be adopted for near-line monitoring systems.

## 1. Introduction

Very low concentrations and/or uneven distribution of pathogens are some of the fundamental challenges associated with food safety testing, clinical diagnostics, and environmental monitoring [1,2,3,4]. Most detection platforms currently on the market cannot accommodate the large sample sizes collected in accordance with regulatory and an industrial standard without subsampling because of factors such as reagent cost, processing time, and space limitations [5,6]. To simultaneously overcome the challenges associated with the criteria for sample collection guidelines and the volume constraints of the sensors used in detection, culture enrichment, sample pretreatment or a combination of both are employed to minimize the likelihood of false negative responses [7,8,9,10]. This has created a need for new testing platforms that are rapid, sensitive, and capable of handling large sample sizes.

Due to their high sensitivity, rapid response, capability to be miniaturized, and adaptability, electrochemical biosensing technology has been applied to the detection of foodborne pathogens [11]. One such method that has the capability for testing large volumes is the flow-through, enzyme-amplified immunoelectrochemical sensor [12,13]. The design of this biosensor incorporates the use of an Ag/AgCl reference electrode, a platinum counter electrode, and a working electrode made from graphite felt. In this system, the graphite felt acts as both the transducer and the capture surface by coating it with target specific antibodies to allow for the selective capture of specific pathogens. Similar to other assays [14], detection is achieved by “sandwiching” targets between capture antibodies and horse radish peroxidase (HRP) labeled reporter antibodies, which culminates in an oxidation–reduction reaction with a chemical substrate that is measured using Osteryoung Square Wave Voltammetry. One advantage to this system is that it can be applied to large volume samples because of the porosity of the graphite felt. The initial study involving the flow-through enzyme-amplified immunoelectrochemical sensor demonstrated its ability to successfully detect *Salmonella enterica* serotype Typhimurium [12], with subsequent work demonstrating the detection of *Escherichia coli* O157:H7 in buffer as well as ground beef homogenates [13]. Encouraging results of this study stated detection limits of 1 × 10^4^ *E. coli* O157:H7 cells using 125 g samples in 1 L volumes of a buffered solution within 3 h. Despite this success, there was difficulty with the matrix clogging the porous electrode and thus significant pretreatment of the sample was required to use this technology in conjunction with ground beef homogenates. To avoid any lengthy pretreatment steps and make optimal use of this detection platform in its current form, food matrices with less particulate matter may be ideal. For example, some of the more fluid food matrices include milk, juice, fruit/vegetable rinsates, and aqueous solutions that may be used in connection with assays of ready-to-eat products.

*Listeria* is found in a range of foods including dairy, meat products, egg products, seafood, freshwater fish, vegetables, and other ready-to-eat (RTE) foods, and can persist and replicate under a wide range of environmental conditions [15]. Currently, 20 different species of *Listeria* have been characterized and they include: *L. aquatica, L. booriae, L. cornellensis, L. costaricensis, L. goaensis, L. fleischmannii, L. floridensis, L. grandensis, L. grayi, L. innocua, L. ivanovii, L. marthii, L. monocytogenes, L. newyorkensis, L. riparia, L. rocourtiae, L. seeligeri, L. thailandensis, L. weihenstephanensis,* and *L. welshimeri.* Although multiple species can be found on food products, *L. monocytogenes* has been found to be the causal agent of human illness. Therefore, for food safety measures, it is important to differentiate *L. monocytogenes* from the other *Listeria* species that may be associated with foods. This is not always a simple task however, especially when trying to distinguish *L. monocytogenes* from *L. innocua.* Due to the similarities amongst the two species, errors can result when using known phenotypic tests [16]. For this same reason and the fact that adverse physiological responses have been noted during antigen challenge, the production of antibodies (which forms the basis for many rapid detection methods) specific for *L. monocytogenes* has also been problematic [17]. The existence of these challenges has created a need for fast and reliable detection methods that can specifically detect *L. monocytogenes* without a reliance upon antibodies or phenotypic differentiation.

Numerous strategies for rapid detection of *Listeria* detection, including electrochemical methods have been extensively studied the literature [18]. Herein, a method for the genetic detection of *L. monocytogenes* is described. The basis for this method involves the capture and subsequent detection of *L. monocytogenes* DNA using small oligonucleotides in a sandwich hybridization format and a previously described flow-through, enzyme-amplified immunoelectrochemical sensor [12,13]. Since antibodies have been the only biorecognition element utilized with the above-mentioned sensing platform, alternative parameters for the incorporation of oligonucleotides have been defined and proof-of-principle shown. Ultimately, this work greatly expands the utility of this platform sensing technology to organisms in which antibodies are not currently available or to targets that can be better differentiated using genotypic as opposed to phenotypic differentiation.

## 2. Materials and Methods

### 2.1. Sourced Materials and Stock Solutions

Electrode polishing suspension and the Ag/AgCl electrodes were sourced from Bioanalytical Systems, Inc. (West Lafayette, IN, USA), platinum wires from VWR (Radnor, PA, USA), and the graphite felt (GF) utilized as the graphite felt electrode (GFE) from Electrosynthesis (Lancaster, NY, USA). NeutrAvidin protein was purchased from Thermo Fisher Scientific (Waltham, MA, USA) and borosilicate beads from Thomas Scientific (Swedesboro, NJ, USA). The 3,3′,5,5′-tetramethylbenzidine (TMB), sodium acetate, glacial acetic acid, sulfuric acid, acetonitrile, Tween-20, and phosphate buffered saline (PBS) tablets were all purchased from Sigma Aldrich (Billerica, MA, USA). PBS tablets were prepared according to the manufacturer’s protocol to yield a 10 mM solution (pH 7.3–7.6). Nanopure water was deionized in-house using a water treatment system (Barnstead, Dubuque, IA). HRP-labeled oligos were manufactured by Bio-Synthesis, Inc (Lewisville, TX, USA) while both modified and non-modified DNA oligos were manufactured by Integrated DNA Technologies (Coralville, IA, USA). 

TKMB buffer was prepared as previously described [19] in 500 mL increments using 10 mM Tris-Cl (pH 8.0), 50 mM KCl, 4 mM MgCl_2_, 200 μg/mL bovine serum albumin (BSA). Saline-sodium citrate (SSC) buffer was purchased as a 20X stock solution of 3 M sodium chloride and 300 mM trisodium citrate (adjusted to pH 7.0 with HCl) from Thermo Fisher Scientific (Waltham, MA USA) and diluted in TKMB. The addition of 1% (*v/v*) sodium dodecyl sulfate (SDS) to 0.1X SSC was used to prepare the SSC/SDS Buffer. 

TMB/H_2_O_2_ solution was prepared freshly for each assay. A 0.3 mM TMB was prepared by diluting a stock solution (6 mg of TMB in 4 mL acetonitrile) in 59.6 mL of 0.20% sodium acetate buffer containing 15 mL of acetonitrile (titrated to pH 4.8–5.0 using acetic acid). Prior to use, 6.3 µL of 3% hydrogen peroxide was added per mL of TMB solution used, with the solution being protected from light until use. Assay stop solution consisted of 1 M sulfuric acid. 

### 2.2. Construction of Flow-Through, Enzyme-Amplified Electrochemical Biosensors

Flow-through, enzyme-amplified electrochemical biosensors were constructed for use throughout this manuscript as described by Capobianco et al. with the following modifications [12,13]. For the electrode preparation, after wetting the 1-inch diameter circle (0.25 inch thick) of GFE with PBS, NeutrAvidin was immobilized on the surface of the GFEs instead of capture antibody as previously described [12,13]. Immobilization of NeutrAvidin enables deposition of any biotinylated moiety, including modified oligonucleotides, to the surface of the GFE. To perform this action, GFEs were immersed in 5 mL of a 7.0 × 10^−7^ M solution of NeutrAvidin in TKMB, which was then flowed through the GFEs. The eluted solution was collected, reapplied to the GFE, and allowed to incubate for one hour. After 1 h, the GFE was rinsed with 10 mL of PBST (0.5% Tween-20 in PBS). The electrode housing for the GFE was prepared in the same manner as that previously described [12,13].

### 2.3. Blocking Solutions

Following the rinse, the GFE was blocked for 30 min with a 5 mL solution of one of the six blocking agents described in Table 1 before being rinsed twice with 5 mL PBST. BSA was purchased from Sigma Aldrich (Billerica, MA, USA) and non-fat milk (Nestle Carnation) from a local supermarket. Since salmon sperm DNA (SSDNA) is known to reduce nonspecific interactions in fluorescent assays [20] and Southern and Northern blotting [21], SSDNA sodium salt from Sigma Aldrich (Billerica, MA, USA) was also tested as described. All shearing of SSDNA was performed via sonication at 40 kHz at room temperature in a Branson 2510 bath (Danbury, CT, USA). Shearing was accomplished with 5 sonication cycles, each with a duration of 30 s followed by a 30 s rest period. The BSA + SSDNA and BSA + SSDNA (sheared) blocking solutions were prepared by first reconstituting the SSDNA to 0.25 mg/mL and then adding powdered BSA to obtain a final concentration of 0.25 mg/mL SSDNA and 0.25 mg/mL BSA.

**Figure 1:**
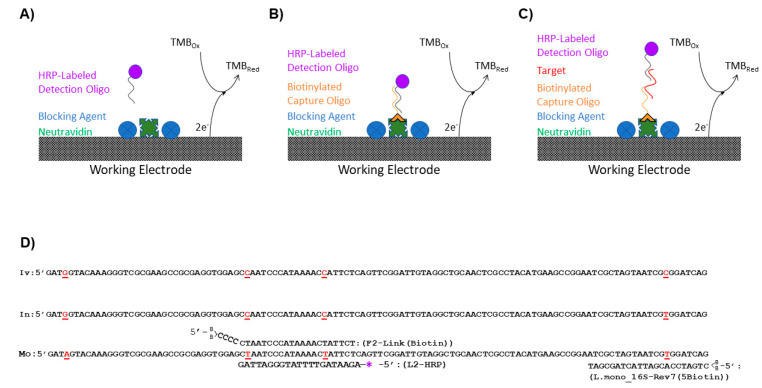
Design of the enzyme-amplified electrochemical biosensor using oligos. (**A**–**C**) Schematic of the different GFE surfaces utilized, with signal generation ultimately relying upon the presence of the HRP-conjugated oligo to facilitate oxidation of the TMB substrate. (**A**) The GFE surface utilized to assess blocking performance. Negative sample preparations assessed aspecific oligo adsorption and positive sample preparations defined the maximum signal intensity. (**B**) The GFE surface utilized for sensor development. The capture oligo binds directly to the detection oligo since it is complementary in sequence. (**C**) The GFE surface utilized for the detection of *L. monocytogenes*. The 16S rDNA sequence from *L. monocytogenes* serves as a bridge binding both the capture oligo and the detection oligo. (**D**) A nucleotide alignment of the 16S rDNA region from *L. ivanovii* (Iv), *L. innocua* (In), and *L. monocytogenes* (Mo). Nucleotides that differed amongst the species are denoted by bold red letters that are underlined. Oligos utilized and their modifications are also shown. The * denotes modification with HRP whereas the$\;{<}_{B}^{B}$ denotes a dual biotin modification.

Negative and positive sample preparations were analyzed for each blocking agent (Figure 1A). Negative preparations determined noise generated from nonspecific binding of the detection L-2 (HRP) oligo to the GFE while positive preparations demonstrated the effects of the blocking reagents on the maximum signal generated. For negative sample preparations, 5 mL of a L-2 (HRP) oligo (Table 2) solutions 4.30 × 10^−9^ M was passed through the GFEs, collected, reapplied to the GFE, and allowed to incubate for 1 h. Following elution, the GFEs were rinsed twice with 5 mL SSC/SDS warmed to 50 °C. For the positive control, a 5 mL solution of TMB/H_2_O_2_ containing 4.30 × 10^−9^ M L-2 (HRP) oligo (Table 2) was made and applied directly to the GFE without rinsing/elution to produce the maximum signal generated by HRP. The reaction with TMB for all samples was allowed to proceed for 20 min in the dark before the addition of 5 mL of a 1 M H_2_SO_4_ stop solution and the electrochemical measurements were recorded using a BAS 100B/W electrochemical analyzer (Bioanalytical Systems, Inc., West Lafayette, IN, USA) in the range of −1200–1200 mV with a sensitivity of 100 mA/V as previously described [12,13]. 

### 2.4. Preparation of the Oligo-Coated GFE Capture Surfaces

The surface of the GFEs for all experiments except those evaluating the blocking solutions was coated with a capture oligo (Table 2) by exploiting the binding of biotin to NeutrAvidin. A 2.0 × 10^−8^ M solution of the biotinylated capture oligo (either F-2 Link (Biotin) (Figure 1B) or L. mono_16S-Rev7 (5Biotin) (Figure 1C) was prepared in TKMB. The oligos were modified with two biotins on the 5′ end of the oligo for increased stability of the biotin/NeutrAvidin bond at the higher temperatures utilized in this assay [23]. The solution was then passed through the NeutrAvidin coated GFEs that were blocked with BSA + SSDNA (sheared) as described above. The effluent was subsequently returned to the vessel containing the GFE and incubated for 1 h. Following the 1-h incubation, the solution was again passed through the GFEs, and the graphite felt electrodes were rinsed 2x with 5 mL SSC/SDS warmed to 50 °C. After rinsing the GFEs were stored at 4 °C until utilized for the experiments described throughout the manuscript. 

### 2.5. Enzymatic Product Production and Detection

To determine the level of nucleic acid strands captured by the GFE, an oligo conjugated with a single HRP enzyme was utilized for its ability to both bind to its complementary oligo sequence and provide a moiety that can be converted into a detectable signal for the sensor. Five milliliters (5 mL) of 9.24 × 10^−9^ M L-2 (HRP) oligo (Table 2) in TKMB was passed through a blocked GFE coated with capture oligos. (Note, experiments with exceptions to the above stated concentration of L-2 (HRP) are described within the method portions that correspond to the specific experiments for which this parameter was changed.) The effluent was collected, reapplied to each respective GFE, and incubated at room temperature for 1 h before being eluted. Following elution, the GFEs were rinsed with 2 × 5 mL SSC/SDS warmed to 50 °C. 

Next, the reaction with TMB was performed and the electrochemical measurements recorded with the BAS 100B/W electrochemical analyzer as described [12,13]. Colorimetric measurements at an absorbance wavelength of 450 nm by a Safire2 plate reader (Tecan Group Ltd.; Männedorf, Switzerland) were also recorded. Note that the absorbance measurements and the electrochemical measurements were conducted on the same day, with the absorbance measurements being conducted first.

Positive controls consisted of a 5 mL solution of 9.24 × 10^−9^ M L-2 (HRP) in TMB/H_2_O_2,_ which was applied directly to the GFE without any subsequent wash/elution steps (Figure 1A). The solution was incubated for 20 min in the dark, after which 5.0 mL of the 1M H_2_SO_4_ stop solution was added. Five minutes following the addition of the stop solution, the electrochemical measurement was recorded. Negative controls received the same treatment as the experimental samples except no biotinylated oligo was applied to the surface of the GFE utilized. It is important to note that throughout the analysis, the GFE associated with both the positive and negative controls were not exposed to any biotinylated oligos. 

### 2.6. Simulated Detection Using Various Detection Oligo Concentrations

**Figure 2:**
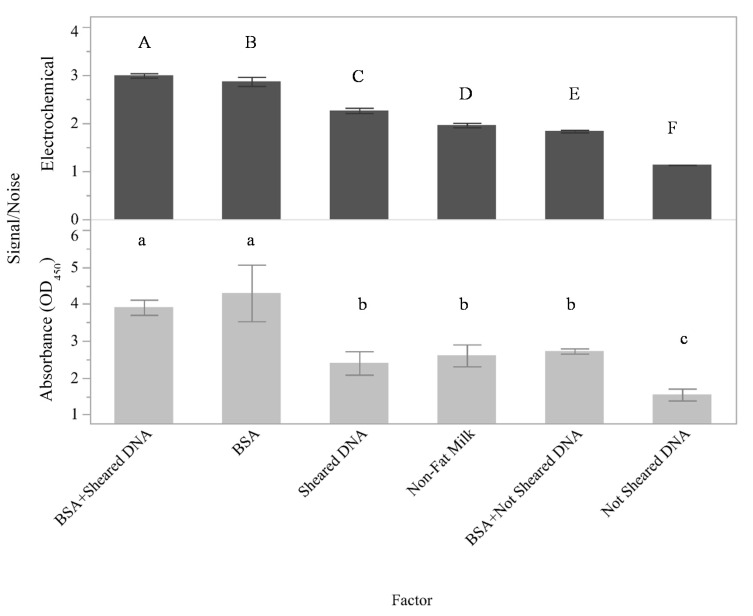
Effects of blocking agents on assay signal. Blocking agents tested are presented as categorical variables along the x-axis while signal-to-noise ratios for both the electrical currents generated using the flow through electrochemical sensor (**top**) and the corresponding absorbance readings at 450 (**bottom**) are presented on the y-axis. The mean of three independent trials is plotted with the error bars representing the standard deviation and statistical differences amongst samples noted by the connecting letters report. Levels not connected by the same letter are significantly different.

For these experiments (Figure 1B), F-2 Link (Biotin) was utilized in lieu of the L. mono_16S-Rev7 (5Biotin) as the capture oligo because it directly binds the HRP labeled L-2 fragment. Capture surfaces were immobilized with F-2 Link (Biotin) (Table 2) and blocked using the procedure described in Section 2.4. Using 5 mL of TKMB, 10-fold serial dilutions of L-2 (HRP) (Table 2) were prepared with concentrations ranging from 1 × 10^−18^ to 1 × 10^−13^ M. The 5 mL solutions were allowed to flow through the GFE once and then reapplied so that it could dwell in contact with the GFE for 60 min. Following exposure, the solutions were discarded, and the electrodes were rinsed once with 10 mL SSC/SDS Buffer, which was warmed to 50 °C. The L-2 (HRP) oligo solution was applied at a concentration of 9.24 × 10^−9^ M and its subsequent detection was performed as described above in Section 2.5 to determine the amount of L-2 captured by the GFE.

### 2.7. Simulated Detection Using 16S Ribosomal DNA (rDNA) Fragments from L. monocytogenes and L. innocua

Initial preparation of the GFEs for the flow-through, enzyme amplified electrochemical biosensor was performed by coating and blocking the electrode surface as described above using Neutravidin and BSA + SSDNA (sheared) blocking solution. The capture ability and specificity of the biosensor were tested using a 20 nM solution of L. mono_16S-Rev7 (5Biotin) oligo (Table 2) in TKMB, which was immobilized onto the NeutrAvidin coated and blocked GFE surface as described above. The electrodes were subsequently exposed to an Ultramer DNA oligo corresponding to the 16S rDNA sequence from either *L. monocytogenes* or *L. innocua* (Table 2 and Figure 1C) by allowing the solution to flow through the GFE once and then reapplying the solution so that it could dwell in contact with the GFE for 60 min. Following exposure to the 16S rDNA fragments, the electrodes were rinsed once with warmed 10 mL SSC/SDS Buffer, the L-2 (HRP) oligo solution was applied, and detection was performed as described in Section 2.6 to determine the amount of *Listeria* rDNA captured by the GFE. 

### 2.8. Detection of Listeria Using Whole Cell Lysates

*L. monocytogenes* F2365 was grown for ~18 h in brain heart infusion media at 30 °C with shaking (180 RPM). The culture was adjusted to an OD_600_ of 1.0 (~10^9^ CFU/mL) with fresh media and 10-fold serial dilutions of the culture were prepared with the 10^7^, 10^6^, and 10^5^ CFU/mL concentrations being utilized for the assay. A 7 µL aliquot of the dilution used was subjected to the 6 × 6 drop plate method to verify the number of cells within the sample [24]. To release the DNA from the live cells, 1 mL of diluted cell culture (either 10^7^, 10^6^, or 10^5^ CFU/mL) was lysed via the OmniLyse Rapid Cell Lysis Kit (Claremont BioSolutions, LLC, Upland, CA, USA) using the manufacturer’s protocol. Whole cell lysates were then brought up to 5 mL in TKMB before being applied to the biosensor.

The GFE for the flow-through, enzyme-amplified electrochemical sensors were prepared as described above for the detection of *L. monocytogenes* 16S rDNA using the BSA + SSDNA (sheared) blocking solution in combination with a 20 nM solution of L. mono_16S-Rev7 (5Biotin) oligo (Table 2) as the capture oligo (Figure 1C). Two rinses of the GFEs containing the immobilized L. mono_16S-Rev7 (5Biotin) oligonucleotide were then performed with 5 mL of SSC/SDS buffer warmed to 50 °C. Electrodes were subsequently exposed to the three dilutions of whole cell lysates of *L. monocytogenes* F2365 using the same apply, flow through, reapply and dwell cycle described above. Post exposure for 60 min, the GFE was rinsed twice with 5 mL of warmed SSC/SDS buffer and the detection oligo was added. Detection using the L-2 (HRP) oligo and the subsequent analysis were also performed as above.

### 2.9. Analysis

Signal-to-noise ratios were calculated by dividing the positive control by the negative control collected within a specific trial. Trials were performed in triplicate and the resulting responses were reported as the average of the three trials. 

The current measured by the BAS 100 B/W was reported at −300 mV as previously described [12,13]. To minimize the variability associated with the batch-to-batch variation of oligo-enzyme conjugate, daily prepared TMB solution, and degradation of hydrogen peroxide, the current of each measurement was divided by the value of the positive control for that trial to normalize the response among trials. The raw data used to generate the normalized responses is presented in the Supplementary Materials associated with this manuscript (Figures S1–S4).

For all data presented, the standard deviation from the means were calculated and are represented by the error bars within the figures. Significance was measured for each set of experiments conducted using Student’s *t*-tests (*p* < 0.05) and statistical differences amongst samples are noted by dissimilar letters.

## 3. Results

The surface area of the graphite felt is extremely large and contains both functional areas for selective binding as well as inactive areas. In food safety diagnostics, the surfaces utilized in testing platforms often come into contact with complex mixtures and may be prone to aspecific and potentially irreversible adherence of mixture components leading to a phenomenon commonly referred to as nonspecific adsorption (NSA). Thus, initial work was conducted to identify conditions that maximized the signal associated with the selective binding of targets to the capture surface while minimizing the signal associated with NSA. The effects of several different potential blocking agents on the assay signal were determined (Figure 1A and Figure 2). Here, signal-to-noise ratios are presented on the y-axis for both the electrical currents measured using the flow through electrochemical sensor and the absorbance at 450 nm.

Although nonfat milk was previously shown to be an appropriate blocking agent for the detection of *Salmonella* and *E. coli* with both buffer and ground beef homogenates [12,13], the present results indicate that nonfat milk is not the optimal blocking agent for assays using oligo fragments. For this assay (Figure 2), the measured signal-to-noise ratio was higher for BSA + SSDNA (sheared) for both the flow-through electrochemical assay (*p* < 0.001) and the absorbance assay (*p* < 0.0001) compared to the other putative blocking agents tested. These data also indicated that while there was a good deal of similarity between the performance of the blocking agents in the two assays, the flow-through electrochemical assay was more sensitive than the absorbance assay to the blocking agent used. This is because the measured responses between all of the experimental factors are statistically significant (0.001 < *p* < 0.003) for the electrochemical measurements, while several of the absorbance measurements (such as BSA + SSDNA (sheared) compared to BSA alone and BSA + SSDNA compared to sheared SSDNA or nonfat milk alone) cannot be differentiated by a Student’s *t*-test with *p* < 0.05. Since the highest measured signal-to-noise ratios were identified to be BSA + SSDNA (sheared) by both the absorbance and electrochemical measurements, BSA + SSDNA (sheared) was selected to be the blocking agent utilized throughout the remainder of this study.

**Figure 3:**
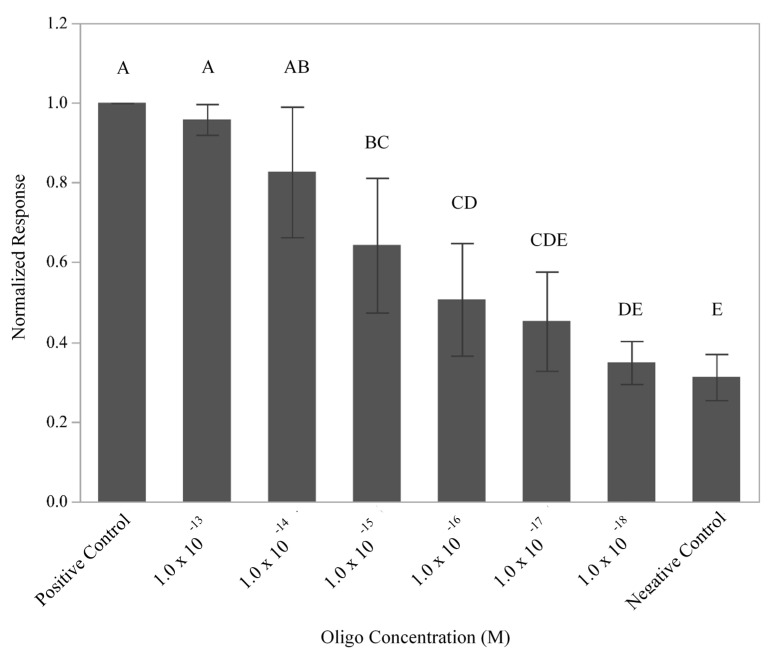
Detection limits for the flow-through, enzyme-amplified electrochemical sensor using oligos as biorecognition elements. The normalized current response was measured for a constant amount of F-2 Link (Biotin) oligo conjugated to the surface of the GFE post exposure to various concentrations (10^−13^–10^−18^ M) of the complementary oligo, L-2 (HRP). The mean of three independent trials is plotted with the standard deviations and statistical differences amongst samples noted by the connecting letters report. Levels not connected by the same letter are significantly different.

Upon identification of an appropriate blocking reagent, an oligonucleotide with known specificity for the 16S rDNA region of *L. monocytogenes* [22] was modified with a single HRP to allow for the detection of DNA from *L. monocytogenes* in the current assay and will be referred to as L-2 (HRP). A nucleotide alignment (Figure 1D) of the 16rDNA region of *L. ivanovii* (GenBank accession # JF967631), *L. innocua* (GenBank accession # S55473), and *L. monocytogenes* (GenBank accession # AE017262) showed that L-2 (HRP) spans two single nucleotide polymorphisms that permitted the differentiation of *L. monocytogenes* from the other closely related *Listeria* species. Another oligo containing the complementary sequence to that of L-2 (HRP), known here as F-2 Link (Biotin), was conjugated to the NeutrAvidin-coated surface of the GFE using the incorporated biotin tag. In this arrangement, F-2 Link (Biotin) served as the capture oligo for the assay and the ability of the flow-through, enzyme-amplified electrochemical sensor to produce a signal when oligos were utilized as the biorecognition element for the assay was ascertained (Figure 1B). To define the limit of detection, signals generated by the flow-through, enzyme-amplified electrochemical sensor using six different dilutions of L-2 (HRP) oligo (10^−18^–10^−13^ M) and a constant amount of F-2 Link (Biotin) (2.0 × 10^−4^ M) were recorded (Figure 3). Comparison of the normalized responses produced by the various concentrations of oligo L-2 (HRP) demonstrated a response that was dependent upon the concentration of L-2 (HRP) with a limit of detection of 1 × 10^−16^ M (*p* = 0.0474). While not all levels were statistically different from one another, the response appeared to follow a linear trend. Using the molarity of the DNA solution, the 5 mL sample volume employed, and the fact that there are six copies of 16S rDNA/cell [25], the total simulated number of cells present was predicted to be ~5 × 10^4^ cells. These results are consistent with previous experiments that used antibodies to detect live and lysed *Salmonella* and *E. coli* cells [12,13]. 

**Figure 4:**
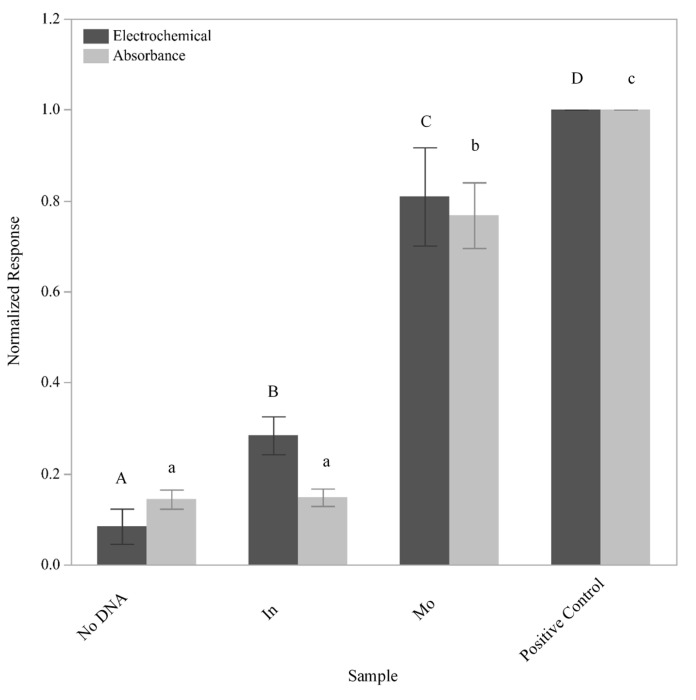
Specificity of the sensor for *L. monocytogenes*. Upon exposure of the sensor to a solution containing fragments of 10^−7^ M 16S rDNA from either *L. innocua* (In) or *L. monocytogenes* (Mo), the resulting normalized responses were determined using both electrochemistry (dark gray bars) and absorbance (light gray bars). Exposure of the sensor to buffer only (no DNA) and the direct application of the detection oligo were used as controls. The mean response of three independent trials is plotted with the standard deviations and statistical differences amongst samples noted by the connecting letters report. Levels not connected by the same letter are significantly different.

In order for the assay to be employed as a detection tool for *L. monocytogenes*, an alternative capture oligo that would not directly bind the detection oligo needed to be assimilated into the assay design. This capture oligo, known as L. mono_16S-Rev7 (5Biotin), bound to a region 43 nucleotides downstream of the L-2 (HRP) oligo and tethered *Listeria* 16S rDNA fragments to the GFE, although it was not specific to *L. monocytogenes* (Figure 1D). However, only tethered *L. monocytogenes* 16S rDNA fragments could ultimately serve as a bridge that enabled detection due to the specific binding of the L-2 (HRP) oligo (Figure 1C). To ensure that all of the conditions necessary for signal generation had been met and that the L-2 (HRP) oligo would be specific for the detection of *L. monocytogenes*, single-stranded Ultramer DNA fragments containing the aforementioned regions were synthesized based upon the known 16S rDNA sequences for both *L. monocytogenes* and *L. innocua* (Table 2). These synthesized fragments were 195 nucleotides in length and were applied individually at a concentration of 10^−13^ M to GFEs that had been both conjugated with the L. mono_16S-Rev7 (5Biotin) oligo and blocked with BSA + SSDNA (sheared). After washing the GFEs with a solution of SSC/SDS warmed to 50 °C to help eliminate nonspecific binding and remove any untethered DNA, electrochemical signals generated by the presence of the HRP were recorded (Figure 4). As a negative control, an identical protocol was followed with the exception that the blocked GFE was not exposed to any additional DNA. The positive control consisted of a direct application of the L-2 (HRP) oligo with subsequent exposure to the TMB substrate (barring all wash steps) to define the maximum signal that could be produced by the amount of HRP presented in the protocol. Student’s t-tests were conducted to compare the signal generated using the different experimental treatments. The response generated post exposure to the *L. monocytogenes* 16S rDNA fragment can be differentiated from the response generated from the *L. innocua* 16S rDNA fragment and the no DNA control by both absorbance and electrochemical responses (*p* < 0.0001). This indicates that the L-2 (HRP) oligo displays specificity for the identified portion of the *L. monocytogenes* 16S rDNA. Given that the response generated with *L. innocua* is significantly different from that generated in the total absence of DNA in the electrochemical assay (*p* = 0.0039) suggests that a low level of cross-reactivity likely exists. In addition, the fact that the absorbance measurements for these same samples was not significantly different (*p* = 0.9025) can be explained by our previous observations that the response obtained via electrochemistry is more sensitive than that obtained via absorbance [12,13]. 

**Figure 5:**
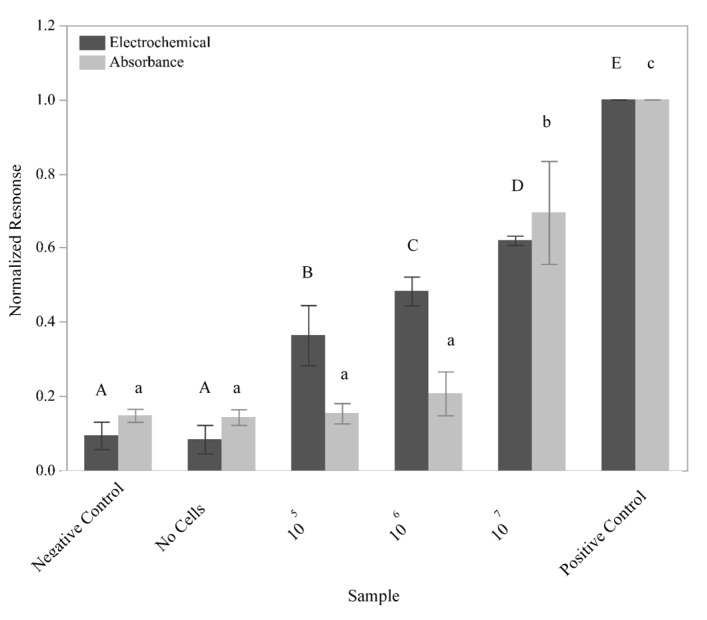
Detection of *L. monocytogenes* from live cells. The normalized response was determined using both electrochemistry (dark gray bars) and absorbance (light gray bars) upon exposure of the sensor to a series of lysed *L. monocytogenes* cells in a 5 mL sample volume. Exposure of the sensor to buffer only (no cells), sensors that did not contain capture oligo (negative control), and the positive control were also performed. The mean response from three independent trials is plotted with the standard deviations and statistical differences amongst samples noted by the connecting letters report. Levels not connected by the same letter are significantly different.

The ability of the sensor to detect *L. monocytogenes* cells was subsequently tested using a 10-fold dilution series of *L. monocytogenes* F2365 (Figure 5). GFEs containing the L. mono_16S-Rev7 (5Biotin) oligo immobilized to the surface were exposed to 10^5^, 10^6^, and 10^7^ lysed cells in 5 mL sample volumes. The number of cells used to inoculate the experimental samples was verified using the 6 × 6 drop plate method (data not shown). The negative control utilized an identical protocol with the exception that the electrode was not immobilized with the L. mono_16S-Rev7 yet was still exposed to 10^7^ lysed cells, which helped to determine the presence of nonspecific binding by the genomic DNA. To test for binding of the detection oligo in the absence of *L. monocytogenes* DNA, a no cells control consisting of a GFE containing the immobilized L. mono_16S-Rev7 (5Biotin) that was not exposed to any cellular material was performed, while the positive control consisted of a direct application of an L-2 (HRP) oligo solution to the GFE as described above.

The electrochemical response generated from the exposure of the GFE to all three dilutions of cells can be differentiated using Student’s *t*-tests (*p* < 0.0076). Unlike the electrochemical response, the response measured via absorbance for the lower dilutions of cells tested could not be differentiated. However, the response generated from the positive control, the negative control, and the sample containing 10^7^ lysed cells were determined to be significantly different (*p* < 0.003). These results once again demonstrated a higher sensitivity for electrochemical as compared to absorbance measurements. 

## 4. Discussion

Blocking agents were a highly important aspect to consider during the development of this electrochemical assay because not only must they help eliminate nonspecific binding onto the GFE but must also allow for the transfer of the signal produced. The most effective blocking agent of those tested was a combination of BSA + SSDNA (sheared) (Figure 2). Neither BSA + SSDNA, BSA alone, or sheared SSDNA alone were as effective, mainly due to a decrease in the measurable signal that could be obtained with those blocking agents. Given that BSA + SSDNA (sheared) resulted in a better signal than BSA + SSDNA, particle size is likely an important factor for proper blocking. Shearing of the DNA via sonication typically yields DNA fragments 50–1500 base pairs (bp) in length with a median around 300–500 bp [26]. It is believed these smaller, sheared DNA fragments not only provide a more uniform coating across the surface of the GFE to prevent nonspecific binding but will also not sterically hinder target DNA strands from accessing their complementary capture oligo in the final design of the sensor compared to DNA that has not been sheared. Based upon molecular weight, the sheared DNA might be expected to occupy more surface area than BSA (sheared DNA molecular weight is ~260 kDa using an assumed size of 400 bp while the molecular weight of the BSA is ~66.5 kDa); however, the BSA may in fact block a larger portion of the surface than expected because of its globular structure compared to the helical structure of the DNA fragments, depending upon the orientation of the DNA. This would explain the increased performance of the BSA alone compared to the sheared DNA alone. It is also interesting to note that for this oligo-based sensor, BSA was shown to be more effective than nonfat milk, which is in contrast to the previous results obtained with the antibody-based sensor [12]. The difference between the measured responses could be due to the presence of neutravidin on the surface of the GFE in these experiments compared to a bare GFE used in prior work. Furthermore, while nonfat milk is a mixture of proteins that has been previously demonstrated to be effective at reducing nonspecific binding in numerous immunoassay platforms, it also contains free biotin which can bind to the neutravidin-coated GFE.

The ability to expand a detection platform beyond the requirement for antibodies can greatly enhance its utility. Not only does this allow for the detection of organisms outside of the scope of the currently available antibodies, but it can also improve other aspects associated with product commercialization as well. For instance, oligos are more stable than antibodies at higher temperatures, which can increase their shelf stability by alleviating the requirement to store them at refrigerated temperatures. Studies have shown that oligos stored at temperatures as high as 37 °C in tris-ethylenediaminetetraacetic acid (TE) remained stable for ~150 weeks [27]. Oligos are also routinely lyophilized, which can further increase their storage potential. In addition, oligos are synthetic and can be manufactured inexpensively. This makes modification of the nucleotide sequence both quick and easy when the detection of alternative targets is desired. Furthermore, hundreds of different chemical modifications are already commercially available for oligos [28], which provides additional options for both signal detection and multiplexing capabilities of the devices themselves. Lastly, because the simple act of heating is known to dissociate complementary strands of nucleic acids, it is possible that detection devices such as this enzyme-amplified electrochemical biosensor can be reusable; further driving down costs for the end user. 

The enzyme-amplified electrochemical assay presented here also has several advantages over other oligo-based methods that may be available for the detection of *L. monocytogenes.* For example, its ability to assay large sample volumes can be vital when dealing with samples with a low incidence rate or uneven distribution of pathogens. Previous studies using this enzyme-amplified electrochemical biosensor have shown that there does not appear to be a significant impact on sensor performance when different volumes are used [13]. In fact, the use of smaller volume samples generally resulted in slightly lower signals than those obtained with larger volume samples when total target number was kept constant. This implies that target concentration may not be an important factor so long as the minimum threshold for detection is achieved. Taken as a whole, the flow-through nature of the device would allow sample volumes to be adjusted accordingly to ensure a sufficient number of targets could be captured. 

Another advantage of this biosensor is that it is not prone to amplification errors that can be seen with other methods utilizing the polymerase chain reaction (PCR) [29] because every capture event in the present assay is an independent event, which eliminates propagation error and can increase the accuracy of the results. Furthermore, as was demonstrated in the present study, only one small oligo (21 nucleotides long) specific for *L. monocytogenes* was needed for the enzyme-amplified electrochemical assay (Figure 1D). This short oligo length may work to improve assay specificity by increasing the match/mismatch ratio. This has been observed previously in oligonucleotide-based DNA microarrays where single nucleotide mismatches are more readily accommodated for energetically by oligos of longer lengths [30]. The need for only a single oligo may also allow for the differentiation of highly similar targets by eliminating the requirement for multiple primers specific to the target of interest and simplify the design of multiplex assays [31].

It is also worth noting that the detection oligo employed in this assay contained only a single HRP enzyme/oligo located at the 5′ end of the oligo. The commercial antibody conjugates used in prior studies display batch-to-batch heterogeneity in the conjugation site, and ratio of antibody-to-ligand which is known to induce variation in measurements [32]. While site-specific antibody conjugation is emerging for drug delivery, this adds significant cost, and there are still associated challenges with scale-up for large scale production. As the site-specific conjugation process can be more easily controlled with oligos, lot-to-lot variation is reduced, and assay repeatability is increased. In addition, the copy number of rDNA in bacterial cells remains relatively consistent across the life of the cell, therefore creating a target that is also consistent for the number of cells assayed. The same cannot be said of most protein epitopes found along the surface of the cell, especially if the cell wall becomes fragmented due to cell lysis, which can result in the presence of various numbers of targets stemming from the original intact cell. Taken together, this suggests that the use of oligos in combination with the enzyme-amplified electrochemical sensor may lead to better quantification of the number of cells initially present in the sample assayed.

The electrochemical assay presented here builds upon the groundwork laid by prior research efforts. While the technology offers some advantages relative to other methods, such as a lower limit of detection [33], is more rapid due to not utilizing bacteria enrichment, [34] and can differentiate *L. monocytogenes* from *Listeria* spp. that are not pathogenic to humans [35]; the technology is not without limitations. For example, the limit of detection is dictated by a finite number of targets present. In order to detect lower concentrations, larger volumes are required, which could lead to increased assay times for situations involving very low concentration of pathogens. From our previous work, we understand that sample volumes up to 1 L can be processed within 1 h; therefore, future work should seek to identify the ultimate volume and flow rate limitations of the sensor. In addition, the assay relies on either 1 or 2 oligos specific to the target and thus, lower specificity can result compared to a real-time PCR assay that incorporates three target-specific oligos. Finally, larger size particulates within the matrix may clog the flow-through device or contain chemicals that inhibit the production and/or transfer of the electrochemical signal [13]. This highlights the importance of continued testing with this device using a variety of different matrices. These limitations are manageable, and further reinforces a point previously phrased by McLamore et al.; that is improved food safety will result from a coordinated approach of applying complimentary sensor systems that utilize the advantages and address the disadvantages associated with the individual technologies [36]. 

## 5. Conclusions

Herein, this investigation presents an oligo-based biosensor capable of the rapid analysis of large (>>1 mL) sample sizes. Though not limited to bacteria, the presented method highlights both its ability to detect *L. monocytogenes*, the only known human pathogen of the genera, and its ability to distinguish it from *L. innocua,* a closely related species that other phenotype-based methods have had difficulty differentiating [16].

This application of flow-through, enzyme-amplified electrochemical detection was effective for detecting both pure oligos in buffer as well as DNA from whole cell lysates. The electrochemical response of the biosensor was benchmarked against an analogous colorimetric approach using spectrophotometric absorbance of the HRP enzymatic product. As has been seen previously, assays based upon electrochemical measurements appear to be considerably more sensitive than the absorbance-based assays. The limit of detection for this biosensor-based assay was revealed to be significantly less than 10^5^ CFU in 5 mL (or <2 × 10^4^ cells/mL) for *L. monocytogenes* cell lysates in a total assay time of approx. 2.5 h, which is comparable to alternative techniques for *L. monocytogenes* detection in foods that cannot accommodate large sample volumes. As this detection platform does not employ a timely culture enrichment step nor require PCR to amplify the number of DNA targets, this approach may find application by regulators and food producers alike for not only testing for the presence of pathogenic *Listeria* in foods, but for hygiene monitoring as well.

## Figures and Tables

**Table 1:** Blocking solutions used during the development of the enzyme-amplified electrochemical biosensor.

Blocking Agent	Preparation
BSA	Reconstituted in TKMB at a concentration of 0.25 mg/mL
Non-fat milk	Reconstituted in TKMB at a concentration of 0.25 mg/mL
Salmon sperm DNA (SSDNA)	Reconstituted in TKMB at a concentration of 10 mg/mL
Sheared SSDNA	Produced via sonication of SSDNA at 40 kHz and subsequently diluted 1:4 with TKMB
BSA + SSDNA	Reconstitution of SSDNA in TKMB [0.25 mg/mL] with the addition of powdered BSA. Final concentrations were 0.25 mg/mL SSDNA and 0.25 mg/mL BSA
BSA + SSDNA (sheared)	Produced in a similar fashion as BSA + SSDNA except sheared SSDNA was utilized

**Table 2:** Oligos used during the development of the enzyme-amplified electrochemical biosensor.

Target and Primer Name	Sequence *	Oligo Modification	Reference
**Capture Oligos**			
F-2 Link (Biotin)	ccccCTAATCCCATAAAACTATTCT	5′ dual biotin	This study
L. mono_16S-Rev7 (5Biotin)	CTGATCCACGATTACTAGCGAT	5′ dual biotin	This study
**Detection Oligo**			
L-2 (HRP)	AGAATAGTTTTATGGGATTAG	5′ HRP	[22]
**Ultramer DNA Oligos**			
L. innocua_16S-Seq (1134–1328)	GACGTCAAATCATCATGCCCCTTAT GACCTGGGCTACACACGTGCTACAA TGGATGGTACAAAGGGTCGCGAAG CCGCGAGGTGGAGCCAATCCCATA AAACCATTCTCAGTTCGGATTGTAG GCTGCAACTCGCCTACATGAAGCCG GAATCGCTAGTAATCGTGGATCAGC ATGCCACGGTGAATACGTTCCC	-	This study
L. mono_16S-Seq (1193–1387)	GACGTCAAATCATCATGCCCCTTAT GACCTGGGCTACACACGTGCTACA ATGGATAGTACAAAGGGTCGCGAA GCCGCGAGGTGGAGCTAATCCCA TAAAACTATTCTCAGTTCGGATTGT AGGCTGCAACTCGCCTACATGAAG CCGGAATCGCTAGTAATCGTGGAT CAGCATGCCACGGTGAATACGTTC CC	-	This study

* Noncomplementary sequences are shown with lowercase letters. - Oligonucleotide does not contain any modifications

## Data Availability

Not applicable.

## Supplementary Materials

The following are available online at https://www.mdpi.com/article/10.3390/s21113754/s1, Figure S1: Comparison of normalized and raw electrochemical data for detection of DNA fragment. The values on the left y-axis correspond to the normalized response while those on the right y-axis are associated with the raw electrochemical signal; Figure S2: Comparison of normalized and raw colorimetric data for the detection of the DNA fragment. The values on the left y-axis correspond to the normalized response while those on the right y-axis are associated with the absorbance signal; Figure S3: Comparison of normalized and raw electrochemical data for detection of DNA from live Listeria monocytogenes cells. The values on the left y-axis correspond to the normalized response while those on the right y-axis are associated with raw electrochemical signal; Figure S4: Comparison of normalized and raw colorimetric data for detection of DNA from live L. monocytogenes cells. The values on the left y-axis correspond to the normalized response while those on the right y-axis are associated with the absorbance signal.
