# Supplementary material for: Flow-Through Electrochemical Biosensor for the Detection of Listeria monocytogenes Using Oligonucleotides
Source: Sensors (Basel). 2021 May 28;21(11):3754. doi: 10.3390/s21113754 (PMC8198859; doi:10.3390/s21113754)
Supplement: Supplementary file 1 [file sensors-21-03754-s001.zip › sensors-1205172-supplementary.pdf]

Supplementary Materials

Flow-Through Electrochemical Biosensor for the Detection of *Listeria Monocytogenes* Using Oligonucleotides

Cheryl M. Armstrong[1], Joe Lee[1], Andrew G. Gehring[1] and Joseph A. Capobianco[1]*

[1] United States Department of Agriculture, Agriculture Research Service, Eastern Regional Research Center, 600 East Mermaid Lane, Wyndmoor, PA 19038, USA

* Correspondence: Correspondence: joseph.capobianco@usda.gov; Tel.: +01-215-233-6447

In our previous work [1], it was noted that extraneous variables affecting the reagents such as lot-to-lot variation and their degradation over time impact amount of product generated. In addition, variations found within and between lots of graphite felt as well as practical variations such as ambient temperature can also affect the values obtained and the ability to compare data from independent trials. To minimize the impact of these variables on our results for each trial we adopted the use of a positive control, which simulates the maximum signal that can be generated. Data within a trial is normalized to the positive control to make comparison between trials more consistent. This topic was discussed in our prior publication [2] and is presented in that work as supplemental information. As with our prior work, colorimetric measurements were utilized as a complimentary technique and ensure similar trends are seen. For transparency, the raw electrochemical measurement data is presented here along with the normalized data discussed in the manuscript.

Normalized and Raw Electrochemical Responses for DNA Fragments

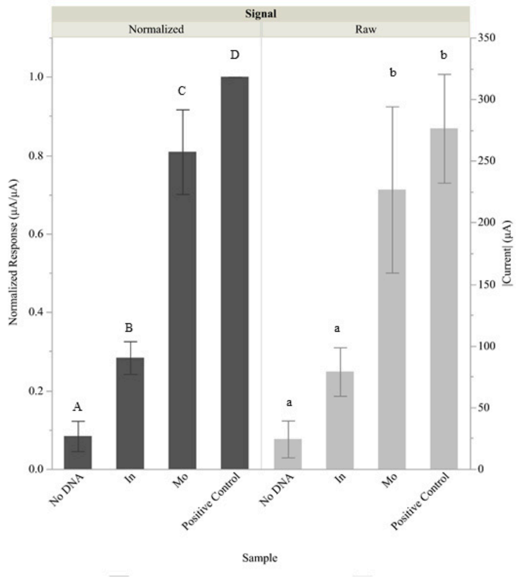

Figure S1. Comparison of normalized and raw electrochemical data for detection of DNA fragment. The values on the left y-axis correspond to the normalized response while those on the right y-axis are associated with the raw electrochemical signal.

Figure S1 presents the raw (light gray bars) alongside the normalized (dark gray bars) electrochemical responses collected for detection of DNA fragments that is presented in figure 4 of the manuscript. Students t-tests were conducted to compare the signal generated using the different experimental treatments. The response generated post exposure to the *L. monocytogenes* 16S rDNA fragment can be differentiated from the response generated from the no DNA control by normalized and raw electrochemical responses ($p<0.0001$ and $p=0.0004$ respectively). The normalized response generated with *L. innocua* is significantly different from that generated in the total absence of DNA in the electrochemical assay ($p=0.0039$), however the corresponding raw measurements cannot be distinguished ($p=0.1509$). While the Students t-test can not statistically differentiate between the levels using the raw data, there does appear to be difference in the responses as the *L. innocua* response is 79±20 μA, while the no DNA response is 24 ±15 μA.

Normalized and Raw Colorimetric Responses for DNA Fragments

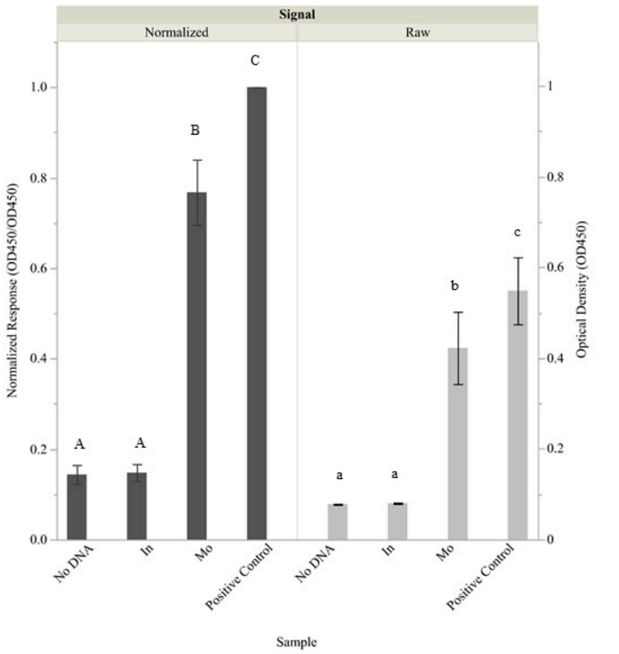

Figure S2. Comparison of normalized and raw colorimetric data for the detection of the DNA fragment. The values on the left y-axis correspond to the normalized response while those on the right y-axis are associated with the absorbance signal.

Figure S2 presents the raw (light gray bars) alongside the normalized (dark grey bars) colorimetric responses collected for the detection of the DNA fragments presented in figure 4 of the manuscript. Students t-tests were conducted to compare the signal generated using the different experimental treatments. The response generated post exposure to the *L. monocytogenes* 16S rDNA fragment can be differentiated from the response generated from the no DNA control by normalized and raw electrochemical responses ($p<0.0001$ and $p<0.0001$). Both the normalized response and the raw colorimetric response generated for *L. innocua* are significantly different from that generated in the total absence of DNA ($p=0.9025$ and 0.9600 respectively).

Normalized and Raw Electrochemical Responses for Listeria DNA

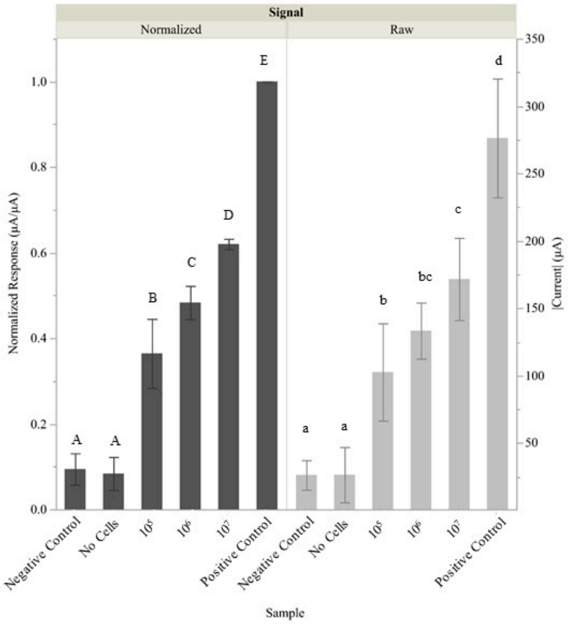

Figure S3. Comparison of normalized and raw electrochemical data for detection of DNA from live *Listeria monocytogenes* cells. The values on the left y-axis correspond to the normalized response while those on the right y-axis are associated with raw electrochemical signal.

Figure S3 presents the raw (light gray bars) alongside the normalized (dark grey bars) electrochemical responses collected for the detection of the DNA fragments presented in figure 5 of the manuscript. Students t-tests were conducted to compare the signal generated using the different experimental treatments. As discussed in the manuscript, each level can be differentiated when the data is normalized, except for the negative control and the case where no cells are present. When the raw data is analyzed, the experimental levels can be differentiated from the negative control and condition that does not have cells ($p<0.01$). However, the ability to differentiate between experimental levels using the raw data is reduced. The sample containing 10^7 lysed cells can be differentiated from 10^5 lysed cells ($p=0.0206$), but 10^7 lysed cells cannot be differentiated from 10^6 lysed cells ($p=0.1582$) nor can 10^6 lysed cells cannot be differentiated from 10^5 lysed cells ($p=0.2504$).

Normalized and Raw Colorimetric Responses for Listeria DNA

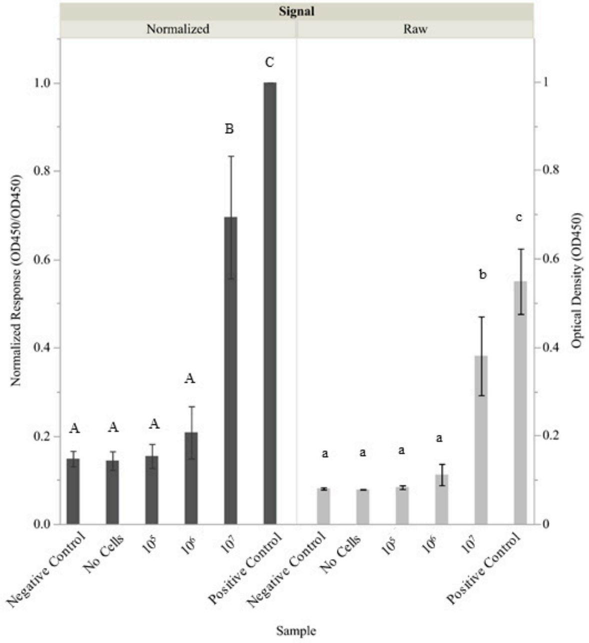

Figure S4. Comparison of normalized and raw colorimetric data for detection of DNA from live *L. monocytogenes* cells. The values on the left y-axis correspond to the normalized response while those on the right y-axis are associated with the absorbance signal.

Figure S4 presents the raw (light gray bars) alongside the normalized (dark grey bars) colorimetric responses collected for the detection of the DNA fragments presented in figure 5 of the manuscript. Students t-tests were conducted to compare the signal generated using the different experimental treatments. As presented in the manuscript, the negative control, no cells, 10^5, and 10^6 lysed cells can not be differentiated from one another but can be differentiated from 10^7 lysed cells and the positive control for the normalized data. The same conclusions are drawn using the raw data.

References

1. Capobianco, J. A.; Armstrong, C. M.; Lee, J.; Gehring, A., Detection of pathogenic bacteria in large volume food samples using an enzyme-linked immunoelectrochemical biosensor. *Food Control* **2020,** 1119 107456.
2. Capobianco, J. A.; Lee, J.; Armstrong, C. M.; Gehring, A. G., Rapid detection of *Salmonella enterica* serotype *Typhimurium* in large volume samples using porous electrodes in a flow-through, enzyme-amplified immunoelectrochemical sensor. *Anal Bioanal Chem* **2019,** 411, (20), 5233-5242.
